# The BE GONE trial study protocol: a randomized crossover dietary intervention of dry beans targeting the gut microbiome of overweight and obese patients with a history of colorectal polyps or cancer

**DOI:** 10.1186/s12885-019-6400-z

**Published:** 2019-12-18

**Authors:** Xiaotao Zhang, Gladys Browman, Wesley Siu, Karen M. Basen-Engquist, Samir M. Hanash, Kristi L. Hoffman, Pablo C. Okhuysen, Paul Scheet, Joseph F. Petrosino, Scott Kopetz, Carrie R. Daniel

**Affiliations:** 1 Department of Epidemiology, Division of Cancer Prevention and Population Sciences, The University of Texas MD Anderson Cancer Center, 1515 Holcombe Blvd, Unit 1340, Houston, TX TX 77030 USA; 2 Department of Medicine, Epidemiology and Population Science, Baylor College of Medicine, Houston, TX USA; 3 Rollins School of Public Health, Emory University, Atlanta, GA USA; 4 Department of Behavioral Science, Division of Cancer Prevention and Population Sciences, The University of Texas MD Anderson Cancer Center, Houston, TX USA; 5 Department of Clinical Cancer Prevention, Division of Cancer Prevention and Population Sciences, The University of Texas MD Anderson Cancer Center, Houston, TX USA; 6 Alkek Center for Metagenomics and Microbiome Research, Department of Molecular Virology and Microbiology, Baylor College of Medicine, Houston, TX USA; 7 Department of Infectious Diseases, Infection Control, and Employee Health, Division of Internal Medicine, The University of Texas MD Anderson Cancer Center, Houston, TX USA; 8 Department of Gastrointestinal Medical Oncology, Division of Cancer Medicine, The University of Texas MD Anderson Cancer Center, Houston, TX USA

**Keywords:** Colorectal cancer survivors, Precancerous colorectal polyps, Dry beans, Obesity, Diet, Gut microbiome, Metabolome

## Abstract

**Background:**

Mouse and human studies support the promise of dry beans to improve metabolic health and to lower cancer risk. In overweight/obese patients with a history of colorectal polyps or cancer, the Beans to Enrich the Gut microbiome vs. Obesity’s Negative Effects (BE GONE) trial will test whether and how an increase in the consumption of pre-cooked, canned dry beans within the context of usual diet and lifestyle can enhance the gut landscape to improve metabolic health and reduce cancer risk.

**Methods/design:**

This randomized crossover trial is designed to characterize changes in (1) host markers spanning lipid metabolism, inflammation, and obesity-related cancer risk; (2) compositional and functional profiles of the fecal microbiome; and (3) host and microbial metabolites. With each subject serving as their own control, the trial will compare the participant’s usual diet with (intervention) and without (control) dry beans. Canned, pre-cooked dry beans are provided to participants and the usual diet continually assessed and monitored. Following a 4-week run-in and equilibration period, each participant provides a total of 5 fasting blood and 6 stool samples over a total period of 16 weeks. The intervention consists of a 2-week ramp-up of dry bean intake to 1 cup/d, which is then continued for an additional 6 weeks. Intra- and inter-individual outcomes are assessed across each crossover period with consideration of the joint or modifying effects of the usual diet and baseline microbiome.

**Discussion:**

The BE GONE trial is evaluating a scalable dietary prevention strategy targeting the gut microbiome of high-risk patients to mitigate the metabolic and inflammatory effects of adiposity that influence colorectal cancer risk, recurrence, and survival. The overarching scientific goal is to further elucidate interactions between diet, the gut microbiome, and host metabolism. Improved understanding of the diet-microbiota interplay and effective means to target these relationships will be key to the future of clinical and public health approaches to cancer and other major diet- and obesity-related diseases.

**Trial registration:**

This protocol is registered with the U.S. National Institutes of Health trial registry, ClinicalTrials.gov, under the identifier NCT02843425. First posted July 25, 2016; last verified January 25, 2019.

## Background

A growing body of research indicates that diet can change the vast community of bacteria (or microbiome) known to affect obesity and cancer risk [1–6]; and presents a potentially effective strategy to improve outcomes among high-risk patients [7]. Provocative findings from controlled human feeding studies reveal that diet-induced changes in the gut microbiome can be both rapid and profound, but easily reversed [8, 9] signifying that beneficial bacteria or microbial communities may need to be continually cultivated to ultimately improve chronic health problems and to prevent latent cancers or their recurrence. Simple strategies are needed for overweight and obese patients who have likely suffered from a history of challenges surrounding food and weight control, as well as for high-risk individuals who prefer dietary approaches to drugs or who cannot tolerate other therapies.

Dry beans (*Phaseolus vulgaris*) are a prebiotic food source rich in bioactive compounds with anti-inflammatory, anti-lipidemic, and chemopreventive properties [10–12]. Despite supportive evidence from parallel obese mouse and human studies [13–17] dry beans are not a particularly popular or well-recognized dietary strategy for reducing the recurrence of colorectal polyps or cancer. One potential barrier to clinical and public health implementation is that none of the previous studies address whether simply increasing or adding beans to the usual diet, as suggested by observational findings in the Polyp Prevention Trial [18], is sufficient to improve gut and overall metabolic health to lower cancer risk. Secondly, the gut microbiome, a potentially transformative tool, has not yet been assessed in a whole dry bean intervention among high-risk, overweight or obese patients with a history of colorectal polyps or cancer.

The BE GONE trial is designed test whether and how a relatively simple increase in canned, pre-cooked dry bean intake can enrich the gut microbiome of overweight/obese patients with a history of precancerous colorectal polyps or colorectal cancer. The trial is also designed to assess whether changes in the gut microbiome precede or parallel changes in other established markers of gut health, metabolic health, and obesity-related cancer risk.

### Hypotheses and objectives

**Fig. 1:**
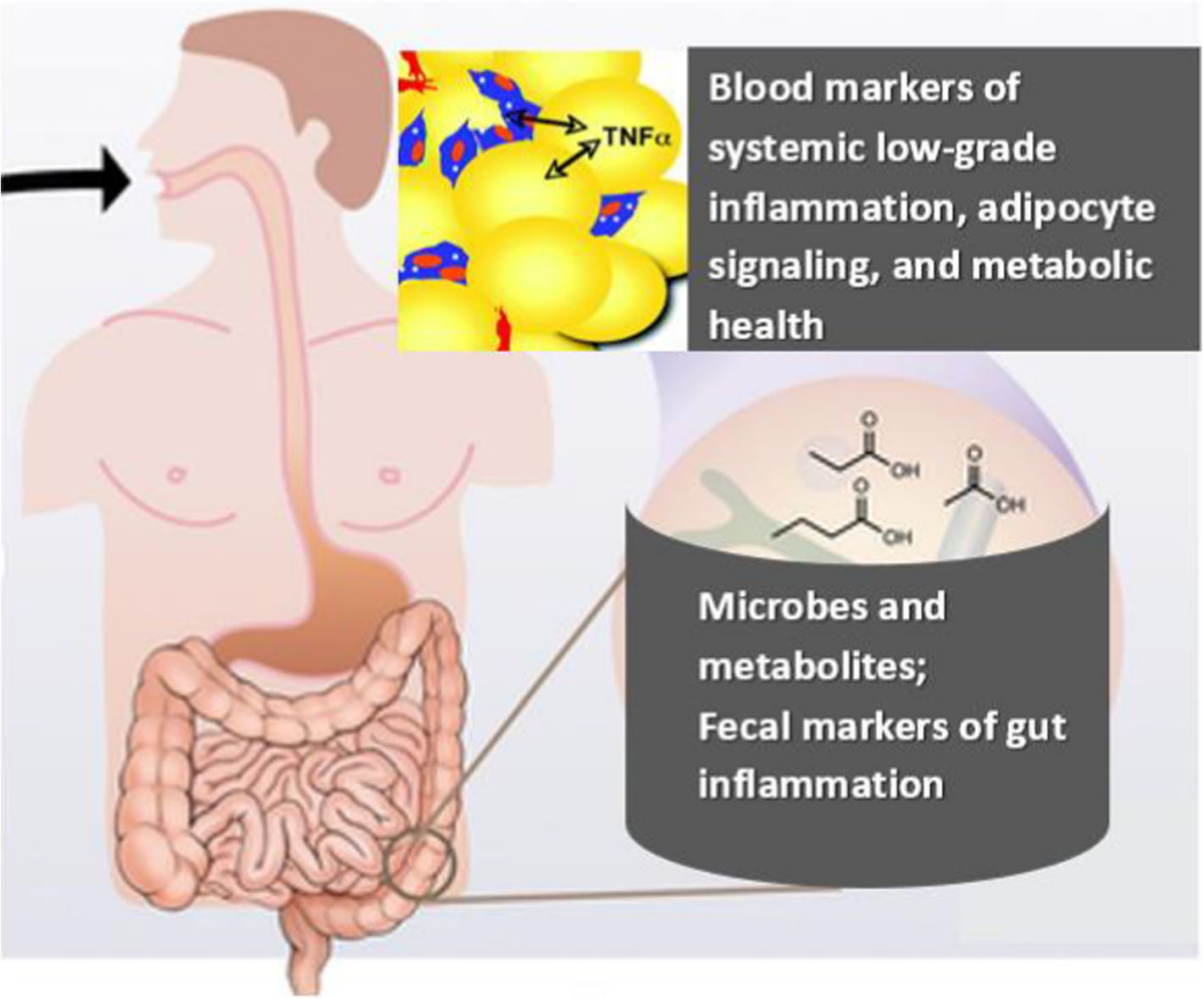
Fiber-rich diet shapes the composition, function and metabolic output of the gut microbiome. Diet modulates host metabolism and inflammation both directly and through the activities of the gut microbiome

Given the prebiotic and antineoplastic properties of beans, and that changes in host diet rapidly alter the composition of gut microbiota, we expect that the dry bean intervention among overweight/obese individuals positive for precancerous colorectal (CR) polyps or CR cancer (herein referred to as “high-risk CR patients”) will enrich or balance the gut microbiome with beneficial bacteria. We further hypothesize that these changes will correlate with improvements in microbial metabolites and host biomarkers that modulate host inflammation and metabolism; and/or lower levels of metabolites positively correlated with obesity-related factors. Characterizing fecal microbiota, blood markers, and metabolites to elucidate interactions between diet, the gut microbiome, and host metabolism will provide insight into overlapping obesity and cancer pathways and more effective methods of upstream dietary prevention (Fig. 1).

### Primary objective

To examine the effect of increased consumption of dry beans on the gut microbiome and blood biomarkers in high-risk CR patients otherwise consuming their usual diet.

### Secondary objective

To develop the field and research procedures of a prospective randomized crossover dietary intervention of whole dry beans, including compliance in our target patient population and the modifying effects of the baseline gut microbiome and usual diet on participant’s response to the intervention.

### Outcome measures

The primary outcome measures are parallel changes in gut microbiota profiles and circulating lipid and adipocytokine profiles from serial stool and fasting blood samples collected at baseline, week 4, and week 8 for each cross-over period. Secondary outcomes include fecal surrogates of gut inflammation and host and microbial metabolites.

## Methods/design

### Overview of trial design

**Fig. 2:**
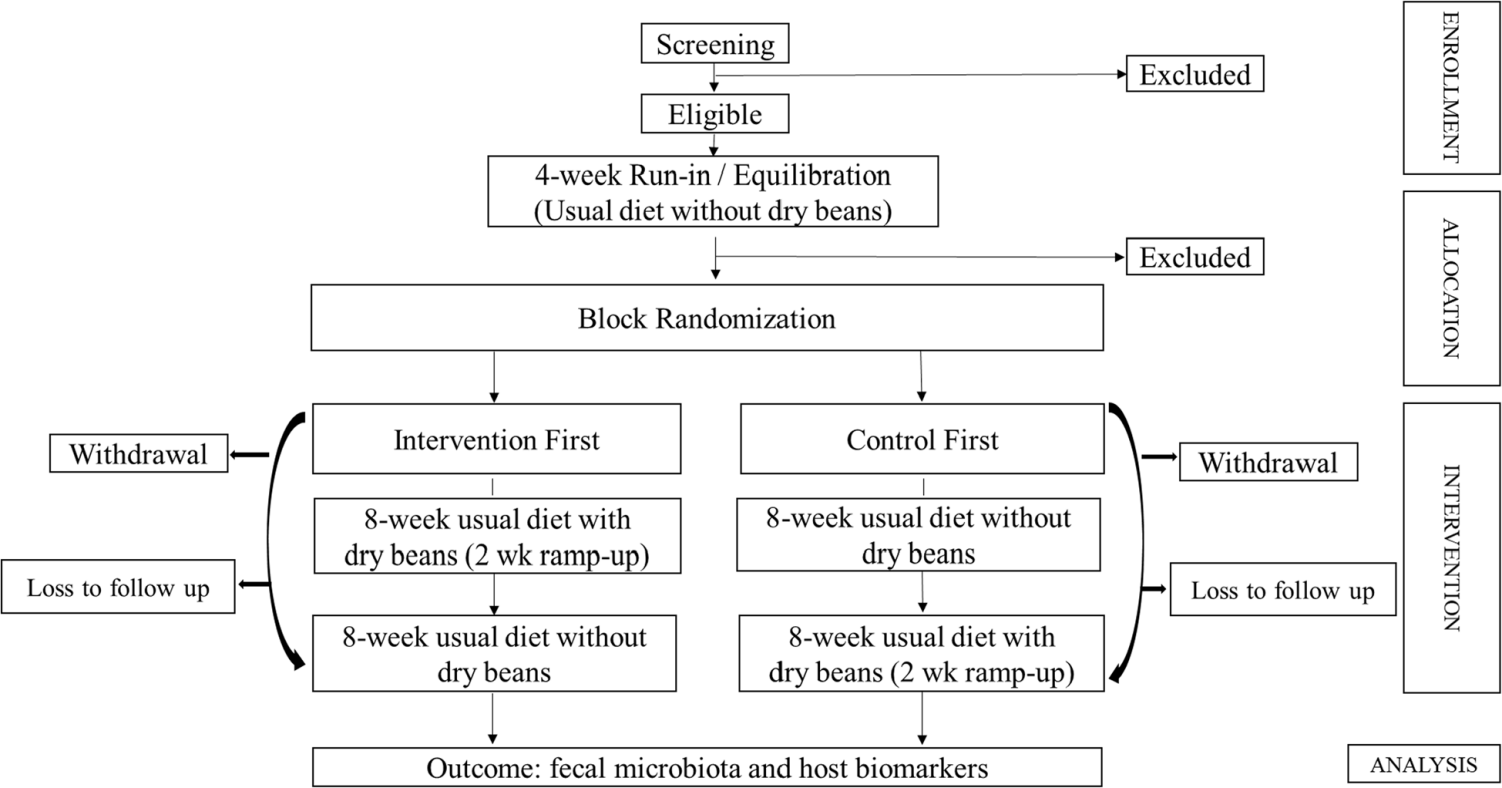
CONSORT flow chart for BE GONE Trial

**Fig. 3:**
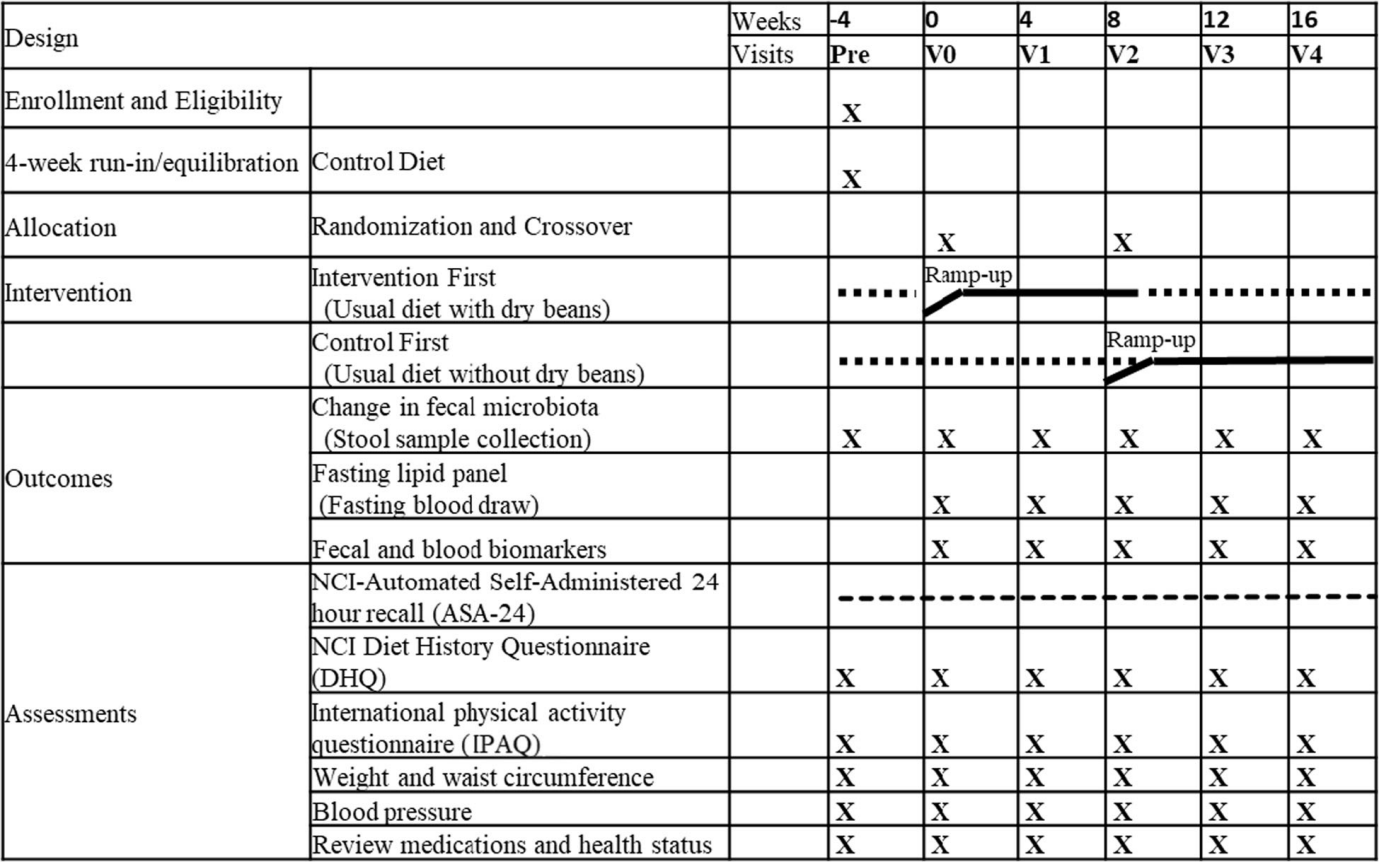
Trial procedures and visit flow

The study is a prospective, randomized, crossover trial (Figs. 2 and 3) of increased dry bean intake added to the participant’s usual diet (Intervention Diet), as compared to the participant’s usual diet excluding dry beans (Control Diet). Following an equilibration (control diet) period, sixty eligible subjects are randomized to one of two diet sequences - Control Diet then Intervention Diet *or* Intervention Diet then Control diet - with each subject acting as their own control. Canned beans are provided during the intervention period and multiple measurements are obtained at baseline and follow-up during each diet period in the sequence. Participants are free-living during the study period and able to choose and prepare their own meals, but are asked to not change any of their other usual habits for the duration of the study. To assess and monitor habitual behavior and adherence, participants are asked to complete web-based assessments at various time points during the study.

### Recruitment and setting

Our main goals are to recruit a diverse set of high-risk CR patients with variation in basal diet and microbiome to address important research questions for future, multicenter trials; and a clinically well-characterized patient population that we can continue to follow over time. The study is conducted at The University of Texas MD Anderson Cancer Center main campus in Houston, Texas. Patients are actively recruited throughout MD Anderson with concentrated efforts in the colorectal screening, treating and survivorship clinics, as well as Kelsey-Seybold, a local referral clinic. Current and former patients are also identified via the institutional tumor registry and electronic medical records. Potentially eligible patients receive invitation emails or postcards with a brief but clear description of the study and procedures. Interested subjects are recruited either during a clinic visit or from inquiry via phone or email.

### Eligibility criteria

Recruitment is targeted to overweight or obese patients with a previous history of colorectal polyps or cancer. Full inclusion and exclusion criteria are included in Table 1.

**Table 1:** BE GONE eligibility criteria

Inclusion Criteria	Exclusion Criteria
• Adult men and women at least 30 years of age • Meet criteria for overweight or obesity via body mass index (BMI) or waist size • Underwent colonoscopy screening within the past 10 years • History of pathology-confirmed precancerous polyp of the colon or rectum; OR colorectal cancer survivor who has completed treatment with adequate maintenance of bowel length (hemicolectomy or low anterior resection) and normalized bowel habits • English-speaking and reside in the greater Houston/outlying areas and/or willing to travel for study-related visits at MD Anderson • Ability to complete web-based dietary assessments twice per week • Willingness to provide stool samples and undergo venipuncture • Willingness to consume/avoid beans as instructed during the 16 weeks from randomization	• Antibiotic use in the past month and unable/unwilling to be deferred to a later recruitment date • Current smoker • Heavy drinker (defined as more than 14 drinks per week) • Currently taking exclusionary prescription medications (including cytokines, immunosuppressive agents, chemopreventive drugs, bile acid sequestrants/selective cholesterol absorption inhibitors) • Regularly taking anti-flatulence medications, probiotics and/or fiber supplements and unable/unwilling to discontinue for the purpose of the study • Major dietary restrictions relevant to the intervention • Total or near total colectomy, greater than 10 cm of small bowel resection • Hereditary colorectal cancer syndromes • Pregnant or lactating or planning to become pregnant

### Informed consent

All subjects must sign informed consent to participate in the study. This consent form fulfills the requirements set forth by the Institutional Review Board (IRB) of MD Anderson. Before signing the consent form, all relevant details and the voluntary nature of the research study, including its purpose, procedures, anticipated risks and benefits are discussed with the potential participant. This dietary study is considered low risk and Data Monitoring Committee exempt. The trial is monitored by the PI and study physicians.

### Study intervention

#### Pre-visit, equilibration and run-in

To establish the basal diet and microbiome and to track compliance with study procedures prior to randomization, eligible and enrolled participants are asked to provide a stool sample and to complete anthropometric, dietary and other assessments during the pre-visit (Fig. 3). In the event that a recent clinical evaluation is not on file for an interested participant, a finger prick glucose test may also be administered. In the subsequent run-in period, participants are asked to follow the control diet (usual diet excluding dry beans) for the first 4 weeks.

#### Randomization and follow-up visits (V0-V4)

Participants who complete the run-in are randomized to begin the intervention diet or to continue the control diet for an additional 8 weeks (Fig. 2). The randomization list was generated by an independent analyst and allocated by a research team member not directly interacting with participants. Participants are block-randomized according to no use vs. regular use of chronic disease medications (namely statins and metformin) commonly prescribed to our target population of overweight/obese high risk CR patients. The randomization visit (V0) and all subsequent trial visits require a fasting blood draw and are conducted in the morning. There are a total of 5 in-person visits every 4 weeks from the randomization visit. At each visit, anthropometry and blood pressure are measured. Medications and changes in health status are reviewed. In addition, a stool sample is brought to each visit (Fig. 3).

#### Ramp-up and dose

Participants are provided with a supply of pre-cooked, canned organic navy beans (*Phaseolus vulgaris*) [19–21] stored in water with sea salt. To examine dose-response and temporal effects and to avoid gastrointestinal (GI) discomfort, participants are counseled to incorporate the canned beans in to their usual diet in a stepwise fashion during the intervention sequence (Fig. 3). Participants begin by consuming ½ cup (1 serving) of beans over the 2 week ramp-up period. At the end of the ramp-up period, participants provide a stool sample by mail. Following the ramp-up, participants consume 1 cup (2 servings) per day for an additional 6 weeks. At week 8, participants who complete the intervention diet crossover to the control diet and vice versa. Two ½ cup servings (260 g) of canned navy beans provide 220 kcal, 14 g protein, 38 g carbohydrate (~ 12% of 2000 kcal diet), 16 g of fiber (~ 64%), 200 mg of sodium (~ 8%), 660 mg of potassium (~ 22%), 12% of daily value for calcium and 20% of daily value for iron.

#### Compliance, adherence, and adverse effects

In addition to in-person visits, the study coordinator and registered dietitian (RD) maintain regular contact with participants via email and phone, while assuring and tracking all study procedures. When on the intervention diet, participants are provided simple, tailored tips and recipes for incorporating the beans in to their usual diet pattern. Participants are asked to keep a “bean log” to record adherence and track GI discomfort. In addition to frequent web-based dietary assessments, the bean log includes a week-by-week daily record of the date, time, amount and meal or manner in which the beans were consumed. Participants are considered adherent if they consume ≥80% of the beans over the intervention period and follow the prescribed regimen on at least 5 days/week. If despite consultation with the RD, some participants still experience difficulties adding the beans to their diet on their own or have specific needs (e.g., air travel), bean crackers prepared by the MD Anderson Cancer Center Bionutrition Research Core kitchen are provided. Participants may also continue the dietary intervention at a reduced/tolerable dose. Of the 30 previous dry bean and CVD trials reviewed, 11 trials reported GI symptoms, such as upset stomach, flatulence, bloating, and increased stool frequency. Across previous similar studies ≤2 participants per trial dropped out due to symptoms [22, 23]. In our study, all adverse events are documented at the time of report, recorded, and reviewed with the study physician. Grade 3 or above adverse effects, according to the Common Terminology Criteria for Adverse Events (CTCAE) Version 5.0, are reviewed immediately with the study physician. If any of the Grade 3 or above adverse effects are deemed serious according to MD Anderson IRB policies, then the IRB is also notified immediately.

#### Withdrawal

Participants may withdraw from the study at any time during the run-in or over the course of the study period (Fig. 2). Reasons for withdrawal are recorded (e.g., intolerance or inability to consume the beans, or a reason unrelated to the intervention). We request that participants, if willing, return a final stool sample by mail.

#### Post-trial follow-up

To explore the post-trial feasibility and impact of diet change, we conduct extended follow-up of all participants who complete the trial to begin to assess whether a long-term increase in bean intake is feasible, maintainable, and/or desirable in our target population. We use both active and passive follow-up methods via email/telephone, and electronic patient records. We assess current dietary habits via the NCI-DHQ (at 6 months and 1-year post-trial completion), as well as health outcomes and results of any subsequent screening exams. Participants may request to cease contact at any time.

### Biospecimen and data collection

#### Stool and fasting blood sample collection

An in-home, fresh-frozen stool sample collection kit, similar to that used in the Human Microbiome Project (HMP) [24] and refined in our previous studies is provided to participants with detailed instructions during the clinic visit. The participant is instructed to collect a stool sample before each scheduled in-clinic visit or return it via pre-paid express mail. Upon receipt, samples are transferred to − 80 °C storage. Fasting blood is collected at each morning in-clinic visit and immediately processed.

#### Diet and lifestyle assessment

Standard computer/web-enabled risk factor questionnaires (REDCap) are used to establish baseline status and monitor deviations over the study period (Fig. 3: Pre, V0, V1, V2, V3, V4). Participant’s diet is continually assessed and monitored throughout the study via biweekly NCI-Automated Self-Administered 24HR (ASA-24) [25] in conjunction with a “past month” web-based NCI diet history questionnaire (DHQ; every 4 weeks) [26]. Physical activity levels are assessed and monitored via validated long- and short versions of the International Physical Activity Questionnaire (IPAQ) [27, 28].

#### Data quality and integrity

All data is stored in a password protected REDCap and MS Access database on a secure and routinely backed-up institutional server. Audits of selected subsets of data are performed to ensure that appropriate safeguards of participant privacy are maintained. Privacy safeguards include appropriate password protection and physical security for all computer systems. Additional quality assurance procedures include a data collection protocol documented in a protocol manual; and a two-stage editing procedure for survey data collection. This two-stage process consists of the initial review of the data collection form by a project member immediately following data collection followed by a second review by a project member who will record any significant deviations from the protocol. Data entry systems, whether via REDCap, scannable forms, or hand entry with verification, specifically provide field checks, range checks for continuous variables and valid value checks for categorical variables; checks for legitimate dates and times and logical consistency. A specific audit trail system that identifies the date, time, and individual making changes on the database is part of the data-entry system. During data collection, we issue reports weekly, or even following any new data entry, depending on the needs of the project or upon the request of the PI.

### Laboratory methods

#### Gut microbiome and metabolome

To examine the impact of a change in dry bean intake within the usual diet on the diversity and composition of the gut microbiome, we will conduct *16S* rRNA gene sequencing on all stool samples collected. Following evaluation of relationships with established blood and stool markers, we will select an informative subset of pre and post-intervention stool samples from participants with marked differences in response to the intervention (e.g., responders vs. non-responders) to conduct whole genome shot-gun (WGS) sequencing and mass spectrometry-based metabolomic profiling of the gut microbiome. This will be complemented by characterization of host pathways via blood-based metabolomics.

#### Microbiome sequencing and data processing

We utilize state-of-the-art methods developed and benchmarked by our collaborating laboratory [29–31]. Briefly, bacterial genomic DNA is extracted and amplified with Illumina barcoded primers and analyzed on the Illumina MiSeq (*16S*) and HiSeq (WGS) platforms. Bacterial mock community samples (QC standards) are routinely included in each run. Alpha and beta (within and between sample) diversity analyses are performed to assess community diversity and richness by calculating the number of observed species for each sample at various sequencing depths. ANOVA and supervised machine learning techniques are used to identify taxa at the level of phylum, class, genus and species that differ significantly in abundance and discriminate between defined parameters. Various clustering algorithms assess whether distinct microbiome clusters or community types are formed.

#### Fecal and serum marker analysis

Fecal surrogates of subclinical intestinal inflammation and gut integrity linked to obesity and CRC risk [32, 33] are measured via established ELISA methods [34]. Fasting serum adipokines and cytokines linked to obesity-driven cancer risk and survival [35–41] and the gut microbiome [42, 43] are assessed via multiplex assays. For comparison with previous dry bean trials a lipid panel (TG, total, HDL, LDL, and VLDL cholesterol) is processed on the day of blood sample collection in a standard manner by a CLIA-certified laboratory.

### Statistical considerations

#### Power

Based on parameters from bean trials of obese individuals that observed comparable changes in LDL cholesterol and inflammatory marker levels [44, 45], with an n of 60 we have > 80% power to detect ≥10% change in LDL levels at a 2-sided α = 0.001 (to account for multiple comparisons). Based on the observed diversity (Shannon Index) and standard deviation (mean 2.5, SD 0.6) among obese polyp patients from our pilot observational study [46, 47], we have > 80% power to detect ≥20% change in microbiome diversity at a 2-sided significance level of alpha = 0.001. For the correlation analyses of changes in the gut microbiome and changes in markers, we have > 80% power at α = 0.001 to detect a significant linear correlation coefficient “r” when the true value is 0.37 [48].

#### Data analysis

Change scores of outcomes will be based on the differences between the beginning and the end of each study period (Figs. 2 and 3); and by subtracting the change experienced over the control period from the change experienced over the intervention period. Paired t-tests will be conducted to assess whether there are significant differences between changes in each subject’s baseline and follow-up outcomes over the equilibration, control, and intervention period. Two-way ANOVA will be used to test for differences in the change scores by categories of other variables. Generalized linear mixed models (GLMMS) will be used to explore potential order or carry-over effects, and to adjust for other potential confounders and assess potential effect modifiers measured at pre-study and study visits. Hierarchically clustered phylotypes will be constructed by the similarity of their dynamics [31] across study periods and subjects and in relation to other variables, such as usual diet pattern.

To examine the effect of a dry bean intervention on the diversity and composition of the gut microbiome, the primary outcome measure will be changes in stool *16S* rRNA gene profiles at baseline and follow-up for each cross-over period. We will quantify the microbial diversity within each subject at each time point; and calculate change scores and construct GLMMs (see above) with repeated measurements (PROC MIXED). We will also construct hierarchically clustered phylotypes by the similarity of their dynamics [31] across study periods and subjects and in relation to other variables, such as usual diet pattern.

To examine the relationship between changes in the gut microbiome and changes in fecal and serum markers, we will follow the procedures described above to quantify changes; and to assess the effects of other variables on these changes. We will assess Spearman correlations of changes in the gut microbiome with changes in fecal and serum markers and employ computational methods to identify taxa associated with differences in serum and fecal markers [49].

To explore relevant functional changes in the gut microbiome we will use a tiered approach. In the first round of analysis of *16S* rDNA data to determine whether and where additional resources should be used for more comprehensive metabolomic and metagenomic profiling, we will use and compare established methods to infer metabolic functional profiles from 16S level data [50, 51]. To assess which compounds are related to which bacteria, we will use a combination of statistical methods and computational tools, including cluster and network analysis coupled with correlation-based non-parametric methods to explore the relationship between host markers and metabolomic pathways, microbial metabolites and gut bacterial species [52]. Principal component and coordinate analyses (PCA & PCoA) will be performed to examine intrinsic clusters within the metabolomic and microbiomic data between the control and intervention diets. In addition, heat maps will be generated using a hierarchical clustering algorithm to visualize the differences within the data set. Differences in gut microbiome composition will be further assessed using a nonparametric test, as described previously [53]. The correlation matrix between the microbial metabolites and gut bacterial species will be generated using Spearman and other correlation methods to explore the functional impact of dry beans on the gut microbiome. For analyses in which the multiplicity of tests is an issue, we will use the false discovery rate to report appropriately adjusted significance levels.

## Discussion

Randomized and controlled, but also “real world” human studies are needed to improve our understanding of the significance of diet-induced changes in the composition and function of the gut microbiome and their multifaceted impact on human metabolic health and obesity-related cancer risk. Gut health requires a fine balance of multiple elements including microbes and their metabolic products (e.g., short chain fatty acids [SCFA]). Dysfunction in any of these components can result in gut dysbiosis linked to obesity and colorectal cancer risk [54–58]. Proposed mechanisms by which prebiotic foods may improve gut health and reduce metabolic complications among obese persons are similarly multidimensional (Fig. 1). This includes increasing populations of beneficial bacteria or functional communities that support intestinal health and barrier function, increasing satiety, and improving lipid or adipocytokine profiles. Whether or not obese patients experience improvements is likely to be strongly influenced by the metabolic output of the microbiota [3, 7]. In addition to promoting high microbial diversity and low pathogen abundance, microbial SCFAs have a major role in maintaining intestinal homeostasis. Locally in the gut they suppress the growth of gram-negative pathogens and function as energy sources for beneficial bacteria, but also have systemic effects on the host, including anti-inflammatory and pro-apoptotic effects [59, 60]. SCFA and in particular, butyrate-producing species are indicators of a diverse, healthy microbiota, and actively involved in maintaining a stable and healthy gut community. However, little is known about dry bean diet-induced, host-microbiota interactions, particularly the effects of differences in individual’s “starting points” with regard to the quality of usual diet and adaptability of the gut microbiome.

Dry beans provide a safe and viable strategy with strong potential for translation and broad implementation. The large PREDIMED study recently reported that higher intake of total legumes (lentils, chickpeas, fresh peas, and dry beans), as assessed by dietary questionnaires, was associated with a 49% lower risk of cancer mortality, an effect that was more pronounced among obese participants (62%) [61]. Dry beans have been tested in multiple CVD risk marker trials [22, 23] and comparatively limited trials in the cancer setting. A recent randomized-controlled trial among colorectal cancer survivors found that a diet enriched with navy bean powder enhanced fecal microbiota and metabolites to modulate metabolic and molecular pathways linked to colon health [17, 62]. The same group reported that navy bean powder was highly feasible to incorporate into meals to increase total fiber intake [63, 64], reaching amounts associated with colorectal cancer chemoprevention and survival outcomes [65].

This study additionally aims to target scientific questions of crucial importance to nutrition and microbiome research and its translation to patients and public health. Given the growing understanding of the complexity of the microbiome, one proffered solution is to challenge it with dramatic changes or high doses to drive physiologically relevant changes. A number of provocative, small, short-term trials and human feeding studies with gut microbiome endpoints testing dramatic and multiple shifts in diet [1, 66–75] have left us with a somewhat limited understanding of “real-world” functional changes in the microbiome that are more reflective of free-living human behavior to inform reasonable and scalable dietary prevention strategies. However, these ground-breaking studies provide a number of important lessons moving forward. Whether or not the target “takes” is largely dependent on the gut microbiota of the host and what the host provides to sustain it in terms of diet. Rapid, profound, and just as easily reversible diet-induced changes in the fecal microbiome similarly induce rapid and notable changes in markers of cancer and CVD risk [1, 2, 70, 76–78]. One implication is that a consistent dietary change would be needed to enrich beneficial bacteria (by fulfilling their nutritional needs) and shape the gut landscape to ameliorate chronic health problems and prevent latent cancers. Patients diagnosed with colorectal polyps or cancer may be initially highly motivated to improve their diets [79–82], but dramatic changes in diet are difficult for most individuals to adopt and sustain; and long-term changes will ultimately be required to impact risk and outcomes in this population. For obese individuals who have consistently struggled with weight and food restriction, small evidence-based changes (such as opening a can of beans) are more likely to be acceptable and ultimately effective.

This study will also generate a sizeable biorepository of serially collected stool and blood samples from clinically well-defined (and followed) high-risk CR patients. Linkage with extensive dietary, as well as lifestyle data collected throughout the trial, will enable us to conduct secondary epidemiologic and biomarker analyses to generate new hypotheses to test in future trials. We will also be able to identify blood-based metabolite biomarkers [83] linked to the fecal microbiome that can be assessed in large prospective cohorts of diet and cancer.

The findings from the BE GONE study will be disseminated through peer-reviewed publications following ICMJE recommendations (http://www.icmje.org/) and presented at international meetings to healthcare professionals. Further dissemination will be through the press and social media. It is expected that findings from the BE GONE trial may inform dietary recommendations and guidelines for high-risk patients and survivors.

## Data Availability

Not applicable. Following publication of the primary analysis, we plan to deposit/make available de-identified data for the purposes of transparency and replication.

## Abbreviations

ANOVA: Analysis of Variance
ASA-24: NCI-Automated Self-Administered 24HR
BE GONE: The Beans to Enrich the Gut microbiome vs. Obesity’s Negative Effects
BMI: Body mass index
CLIA: Clinical Laboratory Improvement Amendments
CTCAE: Common Terminology Criteria for Adverse Events
CVD: Cardiovascular disease
DHQ: NCI diet history questionnaire
g: Gram
GI: Gastrointestinal
GLMM: Generalized linear mixed models
HDL: High density lipoprotein
IPAQ: International Physical Activity Questionnaire
IRB: Institutional Review Board
Kilocalories: Kcal
LDL: Low density lipoprotein
QC: Quality control
rRNA: Ribsomal ribonucleic acid
SCFA: Short chain fatty acid
TG: Triglycerides
V: Visit
VLDL: Very low density lipoprotein
WGS: Whole genome shotgun
